# Early dynamics of photosynthetic *Lhcf2* and *Lhcf15* transcription and mRNA stabilities in response to herbivory-related decadienal in *Phaeodactylum tricornutum*

**DOI:** 10.1038/s41598-020-58885-9

**Published:** 2020-02-06

**Authors:** Shahima Islam, Tanya Sabharwal, Samantha Wu, T. J. Bullock, Mona C. Mehdy

**Affiliations:** Department of Molecular Biosciences, University of Texas at Austin, Austin, TX USA

**Keywords:** RNA metabolism, Transcription, Photosynthesis, Plant stress responses, Marine biology

## Abstract

Abiotic and biotic stresses widely reduce light harvesting complex (*LHC*) gene expression in higher plants and algae. However, control mechanisms and functions of these changes are not well understood. During herbivory, marine diatom species release oxylipins that impair grazer reproduction and serve as signaling molecules to nearby undamaged diatoms. To examine *LHC* mRNA regulation by oxylipin exposure, the diatom *Phaeodactylum tricornutum* was treated with a sublethal concentration of *trans*,*trans*-2,4-decadienal (DD) during the light cycle. Transcriptome analyses revealed extensive suppression of *LHC* mRNAs and a smaller set of up-regulated *LHC* mRNAs at 3 h. For two divergently regulated *LHCF* antennae family mRNAs, *in vivo* 4-thiouracil metabolic labeling was used to distinguish synthesis and degradation rates. Within 3 h of DD exposure, *Lhcf2* mRNA levels and transcription were strongly suppressed and its mRNA half-life decreased. In contrast, *Lhcf15* mRNA mainly accumulated between 3–9 h, its transcription increased and its mRNA was highly stabilized. Hence, DD-treated cells utilized transcriptional and mRNA stability control mechanisms which were likely major factors in the differing *Lhcf2* and *Lhcf15* expression patterns. Widespread *LHC* mRNA regulation and possible effects on photosynthesis may contribute to enhanced fitness in cells impacted by herbivory and other stresses.

## Introduction

In photosynthetic eukaryotes, photosynthetic processes are regulated by extensive internal and external inputs in both unperturbed and stress conditions^1,2^. For example, exposures to high light, nutrient deprivation and herbivory promote mechanisms postulated or demonstrated to maintain homeostasis in photosynthesis^3^. Central to the light reactions are the families of light harvesting complex (LHC) proteins which primarily function to efficiently deliver light energy to photosystem core components^4,5^. Of relevance to this study, a major and widespread gene regulation response to biotic and abiotic stresses in plants including microalgae is a period of extensive repression of *LHC* mRNAs coupled with upregulation of a smaller set of *LHC* mRNAs^6–8^. The functional significance of these changes and the regulatory mechanisms that control them are poorly understood^6,9^. Genes affecting protective light reaction traits, including specific *LHC* genes, represent attractive targets for crop improvement to mitigate adverse stress effects. In tobacco, genetic engineering of photoprotection mechanisms resulted in an accelerated recovery phase from high light stress and a 15% increase in biomass^10^.

Study of photosynthetic acclimation mechanisms in marine diatoms can provide insights for this ecologically and evolutionarily important taxonomic group but also for higher plants and other algal lineages. Diatoms are widely dispersed throughout ocean ecosystems, face rapidly changing conditions and yet are highly competitive and efficient as a dominant phytoplankton group. They contribute almost 40% of marine primary productivity and about 20% of global carbon fixation^11,12^. Diatom LHC proteins all share bound pigments consisting of chlorophylls *a* and *c* and the carotenoid fucoxanthin^13^. Based on phylogeny, there are three major groups of LHC proteins in diatoms: LHCF which are major light harvesting proteins, LHCR which are similar to red algal Photosystem I proteins and LHCX which are stress-responsive LI818/LHCSR-like^14^.

High grazing pressures over long evolutionary time periods have produced distinctive defense strategies among diatoms and other microalgae^15,16^. In the interaction between diatoms and grazers such as copepods and juvenile invertebrates, a well-studied chemical defense strategy in diatoms involves oxylipins which are rapidly synthesized from membrane fatty acids. These compounds are released from damaged cells during grazing^15^ and lysed senescent cells at the end of phytoplankton blooms^17,18^. The concentrations of diatom-derived oxylipins may vary considerably in the environment during herbivory and nutrient stress^19,20^. Oxylipins such as polyunsaturated aldehydes (PUA) or hydroxyacids impaired reproduction including embryo and larvae development in copepods and other invertebrate grazers^21,22^. In a second role, oxylipins released from damaged cells also serve as signaling molecules or toxins to neighboring undamaged cells depending on the type of oxylipin, concentration, and responding diatom species^23^.

Most laboratory studies have employed the PUA class of oxylipins and observed a range of short and long term responses in undamaged diatoms. Responses occur to PUAs even in diatoms which produce different classes of oxylipins, such as *Phaeodactylum tricornutum* which shows herbivory-induced production of non-volatile aldehydic acids such as oxo-acids 12-oxo-(5Z,8Z,10E)-dodecatrienoic acid (12-ODTE) and 9-oxo-(5Z,7E)-nonadienoic acid (9-ONDE) that inhibit normal development of invertebrates^24^. For example, high concentrations of the PUA *trans*,*trans*-2,4-decadienal (DD) triggered an apoptosis like pathway and were lethal as shown for *Thalassiosira weissflogii*^25^ and *Phaeodactylum tricornutum*^26^. A recent study examined the effects of DD concentration on redox status of the chloroplast, nucleus and mitochondria using organelle-specific expression of a redox-sensitive GFP in *P. tricornutum*^27^. High lethal DD concentrations caused striking oxidations of the redox-sensitive GFPs while sublethal 5 µM DD resulted in no detectable elevated oxidation in all three organelles throughout the 3.8 h interval. In contrast, low sublethal concentrations of PUAs elicit responses which may contribute to cell health and survival. Responses measured in various diatoms include transient reductions of growth^28^, reduced adhesion to substrates within 4 h of DD exposure^29^, and increased synthesis of carotenoid compounds, involved in the xanthophyll cycle and potentially having antioxidant functions, which began within 1 h of PUA exposure^30^. In *P. tricornutum*, the organism of the present study, pretreatment with a sublethal concentration of DD for 2–3 h greatly increased viability after treatment with a subsequent lethal concentration^26,31^. In addition, cells exhibited extensive membrane lipid remodeling and altered membrane permeability properties within 3 and 6 h of sublethal DD exposure^31^.

The effects of sublethal PUA treatments on LHC gene expression and photosynthetic efficiency have been measured in several diatom species. In *P. tricornutum* exposed to sublethal DD treatment for 6 h, genome-wide EST studies revealed that all 23 detected *LHC* mRNAs were downregulated except for *Lhcf15* which was upregulated^32^. Yet, *P. tricornutum* cells grown under constant light conditions and exposed to sublethal DD maintained stable photosynthetic efficiency of PSII for 6 days^33^. Similarly, cells of the diatom *Skeletonema marinoi* treated with sublethal PUAs were not impaired in PSII photosynthetic efficiency^30^. Control of mRNA levels from endogenous genes by transcriptional and/or mRNA decay mechanisms has not been determined for *LHC* or other mRNAs in diatoms to our knowledge. Limited studies exist defining these mechanisms to achieve *LHC* mRNA regulations in higher plants and other algae. In rice subjected to drought or salt stress, reduced light harvesting *Cab1* mRNA levels were due to decreased mRNA stability^34^. In *Chlamydomonas reinhardtii* during high light stress, decreases in two *LHC* mRNA levels were due to repressed transcription and reduced mRNA stability, respectively^35^.

The current study focused on *LHC* gene expression in undamaged *P. tricornutum* cells exposed to sublethal DD shortly after light onset in the diurnal cycle. After overall *LHC* transcriptome analysis during an early 6 h response period, we used metabolic labeling with 4-thiouracil (4-TU) to measure transcription and mRNA decay rates for two divergently expressed *LHC* antennae genes, *Lhcf2* and *Lhcf15*, from the major class called *LHCF*. Use of multiple time points over most of the light period enabled resolution of changing mRNA expression control mechanisms between DD stimulated and control cells.

## Results

### DD alters *LHC* transcript levels during the light period in *P. tricornutum* cells

**Figure 1:**
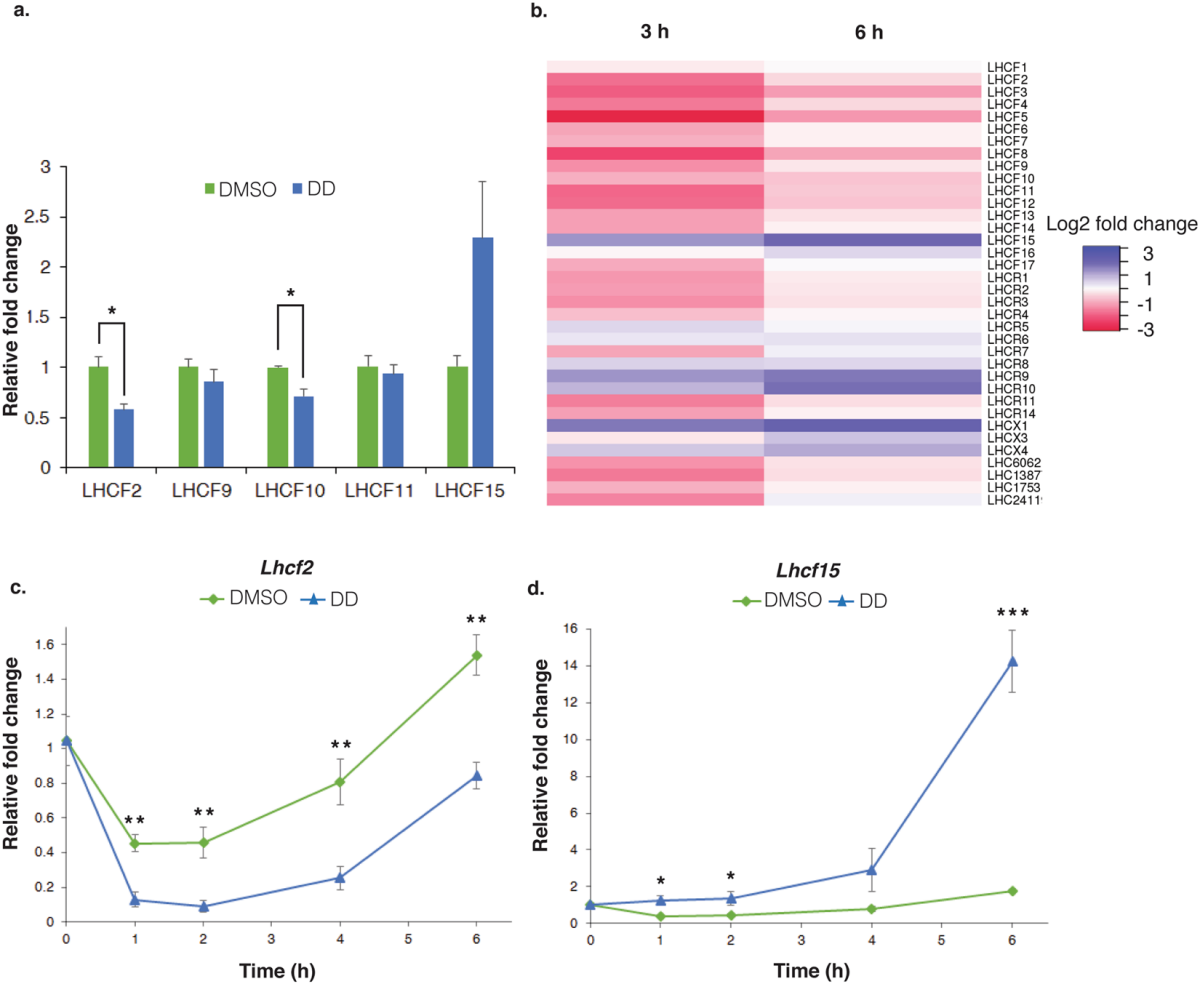
Effects of DD on *LHC* gene expression. Cultures were treated at 0 h with 10 µM DD or 0.1% DMSO as final concentrations. **(a)** Quantitative RT-PCR (qRT-PCR) analysis of *Lhcf2*, *Lhcf9*, *Lhcf10*, *Lhcf11* and *Lhcf15* expression at 6 h after DD treatment. Cell density 8.0 × 10^5^ cells/ml, n = 3 biological replicates. **(b)** Heat map analysis of *LHC* gene expression at 3 and 6 h after DD treatment. Color key indicates expression levels. Cell density 3–3.2 × 10^6^ cells/ml, n = 2 biological replicates. qRT-PCR analyses of *Lhcf2* **(c)** *Lhcf15* **(d)** mRNA kinetics. Cell density 2.5 × 10^6^ cells/ml, n = 5 biological replicates. Reference gene was *TBP* (TATA-box binding protein). Fold changes are relative to 0 h. Relative fold change = mean ± SEM. Error bars not appearing are merged into the time points. *P < 0.05, **P < 0.01, ***P < 0.001, two-tailed student’s t-test.

To initially compare DD-regulated *LHC* gene expression in our *P. tricornutum* cultures to previous EST database findings^32^, four *LHCF* mRNAs and *Lhcf15*, shown to be down- and upregulated, respectively, were examined by RT-qPCR. Cells were treated with 10 µM DD which was shown as sublethal based on cell viability and growth curves^31^. In cells treated with DD for 6 h, *Lhcf2* and *Lhcf10* mRNAs were significantly downregulated to 0.58 and 0.71 fold, respectively, compared to the DMSO solvent control (Fig. 1a). *Lhcf15* mRNA was upregulated while *Lhcf9* and *Lhcf11* mRNA levels remained about the same. These DD induced mRNA regulations were similar to those presented in the EST database except for the last 2 transcripts which were also downregulated in the database^32^.

To investigate DD effects on *LHC* mRNAs further on a genome-wide scale, RNA-Seq data were analyzed at 3 and 6 h of DD or DMSO exposure. The heat map presents the expression of 36 nuclear encoded *LHC* transcripts (Fig. 1b) and the log2 fold changes compared to the DMSO controls are given in Supplementary database Table S1. At 3 h, DD treatment strongly suppressed 15 of 17 *LHCF* transcripts (−0.2 to −3.1 log2 fold) and 7 of 12 detected *LHCR* transcripts (−0.77 to −1.6 log2 fold) (Padj < 0.001). All four unclassified *LHC* transcripts were downregulated (−0.99 to −1.6 log2 fold). At 6 h, the majority of these mRNAs were restored and not significantly different from the DMSO control levels. Nine *LHCF* and 2 *LHCR* transcripts remained significantly downregulated with a similar trend of smaller magnitude differences from the control (−0.44 to −1.3 log2 fold for *LHCF*; −0.13 and −0.43 log2 fold for *LHCR*) (Padj < 0.001 to 0.05). In contrast, several *LHCF* and *LHCR* mRNAs exhibited a different pattern and were significantly upregulated at 3 h with levels further increased at 6 h. Alone among the *LHCF* group, *Lhcf15* mRNA increased from 1.3 to 1.97 log2 fold. *Lhcr*9 and *Lhcr*10 mRNA levels also increased from the 3 h to the 6 h levels (1.3 to 1.6 log2 fold and 0.90 to 1.75 log2 fold, respectively). Lastly, all 3 transcripts identified from the 4 *LHCX* genes, *Lhcx1*, *Lhcx3* and *Lhcx4*, were significantly upregulated at 6 h (0.7 to 2.1 log2 fold). *Lhcx1* and *Lhcx4* mRNAs were also significantly upregulated at 3 h (1.6 and 0.6 log2 fold, respectively) and accumulated to higher levels at 6 h. Overall, *LHC* transcript regulation by DD fell into two main groups: a larger group of mRNAs that exhibited early, transient downregulation and a smaller set of upregulated mRNAs exhibiting increasing upregulation over the period studied.

We chose two *LHCF* members to explore the mechanisms of divergent photosynthetic *LHC* transcript control in response to DD. Specifically, *Lhcf2* was chosen on the basis of its significant downregulation by DD treatment and the relatively extensive knowledge of its diurnal regulation and gene regulatory regions^36,37^. *Lhcf15* mRNA was the sole upregulated *LHCF* transcript. *LHCF15* mRNA is of interest as it was induced by prolonged dark and red light^14,38^ and encodes a distinctive red-shifted absorbing protein^39,40^. To resolve the kinetics of DD regulation during the light cycle, a more detailed time course was carried out and fold changes relative to the 0 h time point are presented (Fig. 1c,d). In DMSO treated cells, *Lhcf2* transcript was downregulated through 2 h then gradually increased to 0.8 fold at 4 h and 1.53 fold at 6 h (Fig. 1c). In DD treated cells, a similar pattern was observed but *Lhcf2* mRNA levels were significantly lower at all time points. For example, *Lhcf2* mRNA levels in DMSO treated cells were 0.45–0.46 fold through 2 h while they were 0.1–0.09 fold in DD treated cells. For *Lhcf15*, transcript levels in DMSO treated cells downregulated to 0.3 fold at 1 h, then gradually increased to 0.4 fold at 2 h, 0.8 fold at 4 h and 1.7 fold at 6 h (Fig. 1d). In DD treated cells, *Lhcf15* mRNA initially slowly increased to 1.2 fold at 1 h and 1.3 fold at 2 h, then rapidly upregulated to 2.9 fold at 4 h and 14 fold at 6 h (all time points significant except 4 h). The kinetic analysis revealed early onset (within 1 h) and sustained suppression of *Lhcf2* mRNA by DD treatment yet appearing to parallel the natural light cycle regulation. In contrast, DD treatment resulted in an early, modest increase in *Lhcf15* mRNA level with strongest enhancement occurring late, between 4–6 h.

### 4-Thiouracil (4-TU) in culture media was efficiently incorporated into *P. tricornutum* RNA

**Figure 2:**
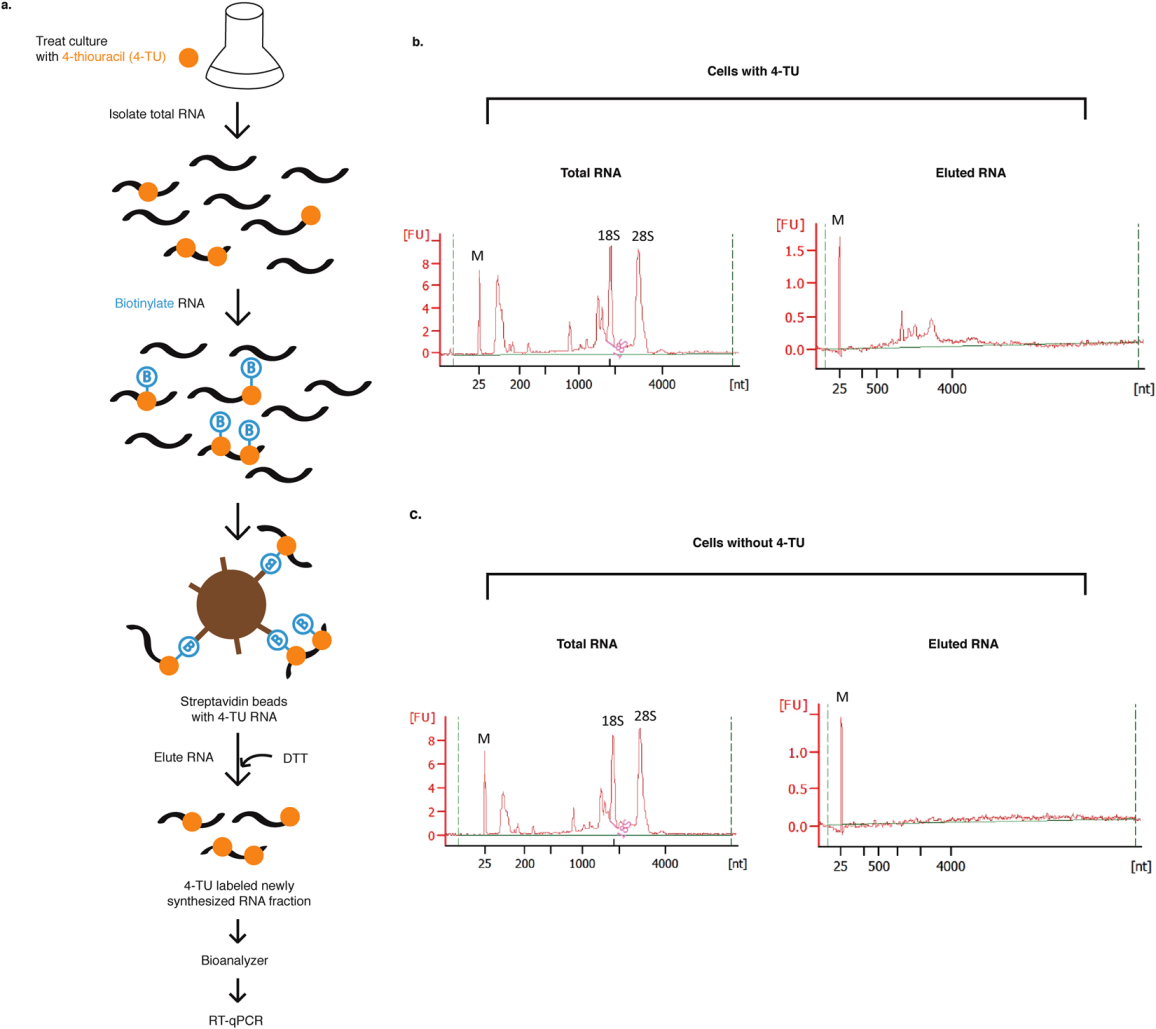
*In vivo* incorporation of 4-TU into RNA of *P. tricornutum*. (**a**) Schematic workflow of 4-TU labeling into RNA and detection. Bioanalyzer profiles of RNA fractions from cell cultures treated with 0.5 mM 4-TU (**b**) or 0.1% DMSO as solvent control (**c**) for 1.5 h. From each treated culture, total RNA represents the biotinylated RNA fraction and eluted RNA represents the newly synthesized RNA fraction. Equal masses of biotinylated RNA fractions were used to prepare the eluted RNA fractions.

The observed changes in the *Lhcf2* and *Lhcf15* mRNA levels in response to DD could be regulated either at the level of transcription, mRNA stability or both. *In vivo* metabolic labeling of RNA with 4-thiouracil (4-TU) or 4-thiouridine is an efficient method to isolate labeled newly synthesized RNA fractions for determining mRNA synthesis and decay and has been developed in a range of eukaryotes^41,42^. We used 4-TU to label newly synthesized RNA because *P. tricornutum* contains the gene for uracil phosphoribosyltransferase (Uniprot ID B7FUW5), an enzyme for recycling uracil to uridine monophosphate in the pyrimidine salvage pathway^43^. Initially, we assessed cell growth in media containing 4-TU (1–3 mM) over several generations. While 1–3 mM 4-TU slowed growth is a dose dependent manner, 0.1 and 0.5 mM 4-TU treatments did not reduce growth compared to DMSO control cells (Supplementary Fig. S1). In addition, an Evans Blue test of cell viability confirmed live cell percentages were similar between 0.5 mM 4-TU and DMSO control cells for 1.5 and 3 h (Supplementary Table S2). Furthermore, incorporation of the label into total RNA was assessed through a dot-blot (Supplementary Fig. S2). RNA from cultures treated with 4-TU or solvent (No 4-TU) were biotinylated as described in Fig. 2A to enable detection of 4-TU in the samples. Signals were stronger in RNA from 0.5 mM compared to 1.0 mM 4-TU treated cells and both were stronger than the signal in the no 4-TU treated cells RNA, the latter likely due to residual free biotin. Taken together, 0.5 mM 4-TU was considered as an effective concentration for subsequent metabolic labeling experiments.

To verify efficient isolation of 4-TU labeled newly synthesized RNA fraction from unlabeled RNA, cultures were treated with and without 4-TU (DMSO solvent control) for 1.5 h. Figure 2a shows the steps for the 4-TU culture RNA processing and the same processing was applied to untreated culture RNA. Purified RNA was subjected to biotin-HPDP which covalently reacted with the newly synthesized 4-TU tagged RNA. The biotinylated total RNA was then fractionated into newly synthesized RNA fractions through streptavidin magnetic beads. Bioanalyzer profiles of 4-TU labeled and unlabeled total RNA fractions showed intact rRNA peaks (Fig. 2b,c left). Whereas, the eluted 4-TU labeled newly synthesized RNA fraction showed a broad range of RNA size distributions and was free of visible rRNA bands indicating that the pool mostly consisted of mRNAs (Fig. 2b right). Bioanalyzer profiles of eluted RNA fractions from cells without 4-TU showed undetectable peaks indicating very low mass of RNA fraction (Fig. 2c right). Hence, 4-TU labeled newly synthesized RNA fractions were effectively separated from unlabeled RNA fractions in *P. tricornutum* for the first time.

### *Lhcf2* and *Lhcf15* mRNA stabilities were altered in the presence of DD

**Figure 3:**
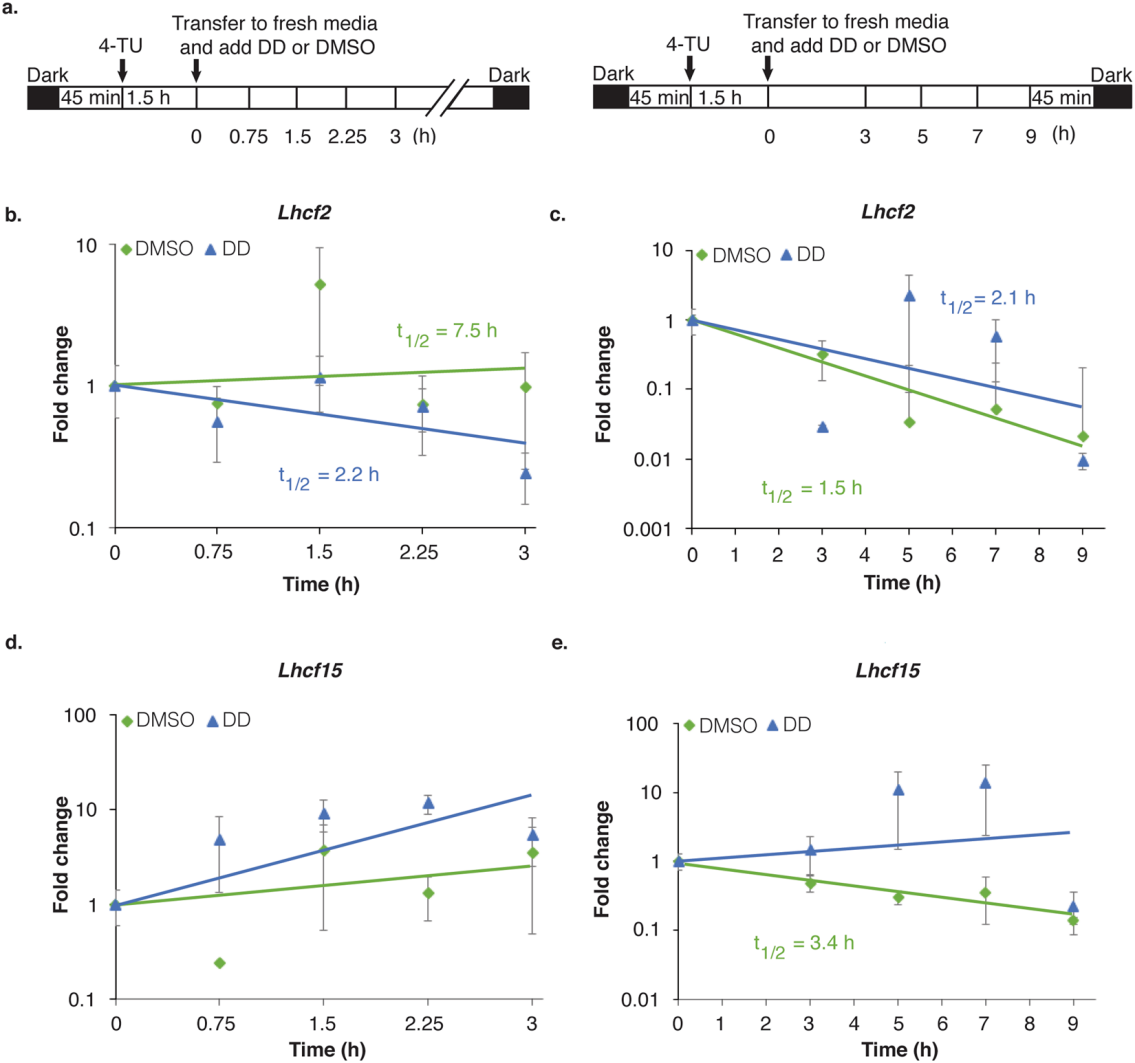
Effects of DD on *Lhcf2* and *Lhcf15* mRNA stabilities. **(a)** Schematic representation of 3 h (left) and 9 h (right) 4-TU pulse-chase experiments. Numbers below the bars indicate harvest times after DD and DMSO treatments. Calculated mRNA half-lives are given inside the graphs. RT-qPCR analyses to determine newly synthesized *Lhcf2* mRNA stabilities, up to 3 h **(b)** and up to 9 h **(c)** after treatments. RT-qPCR analyses to determine newly synthesized *Lhcf15* mRNA stabilities, up to 3 h **(d)** and up to 9 h **(e)** after treatments. Cell density 2.5 × 10^6^ cells/ml, n = 3 biological replicates. Fold changes are relative to 0 h. Fold change = mean ± SEM. Error bars not appearing are merged into the time points. *P < 0.05, **P < 0.01, ***P < 0.001, two-tailed student’s t-test.

Stability of mRNA is a key regulator affecting mRNA levels^44^. In order to determine mRNA decay rates in DD and DMSO treated cells, we optimized 4-TU pulse-chase experiment conditions. Initially, pulse-chase experiments were performed with 1.5 h of 0.5 mM 4-TU pulse labeling and 10 mM uracil, 5 mM uridine or f/2 media alone chases. In both uracil and uridine chases, cells acquired darker colors and exhibited much slower filtration rates during harvesting than cells exposed to fresh f/2 media chase which were unaffected (Supplemental Fig. S3). So, f/2 medium was used as the chase method to avoid physiological perturbations. Figure 3a shows the experimental set-ups for 4-TU pulses and short 3 h and long 9 h chase periods in the presence of DD or DMSO. RT-qPCR analyses of *Lhcf2* and *Lhcf15* mRNA levels from 4-TU labeled biotinylated total RNA fractions in the pulse-chase experiments verified DD and DMSO effects on mRNA regulations as previously shown (Supplemental Fig. S4). Successful 4-TU pulse-chase was confirmed from mono-exponential decline of 4-TU labeled newly synthesized RNA concentrations resulting from equal masses of biotinylated RNA fractions (Supplemental Fig. S5).

In the 3 h pulse-chase experiment, in DMSO treated cells, *Lhcf2* transcript half-life was 7.5 h while in DD treated cells it was 2.2 h indicating DD exposure rapidly destabilized this transcript (Fig. 3b). In the 9 h pulse-chase, the half-life strikingly decreased to 1.5 h in the DMSO treated cells while in DD treated cells it was 2.1 h. Thus, *Lhcf2* half-lives were comparable in the DMSO and DD conditions later in the light cycle or there was a slight stabilization in the DD condition (Fig. 3c).

In the 3 h pulse-chase experiment, *Lhcf15* transcript at 3 h DD exposure period was strongly stabilized in DD condition compared to the DMSO treated cells but precise mRNA half-lives were not possible to measure for either treatment conditions (Fig. 3d). Most probably, residual 4-TU incorporated into the cells were not chased completely by fresh f/2 media within 3 h. At 9 h pulse-chase, the half-life of *Lhcf15* transcript in DMSO treated cells was 3.4 h while in DD treated cells it was highly stabilized and still indeterminate (Fig. 3e). Overall, DD induced changes in both *Lhcf2* and *Lhcf15* mRNA decay rates were consistent with mRNA decay regulation as a substantial factor influencing the observed DD effects on their steady-state mRNA levels.

### DD regulated both *Lhcf2* and *Lhcf15* transcription rates

**Figure 4:**
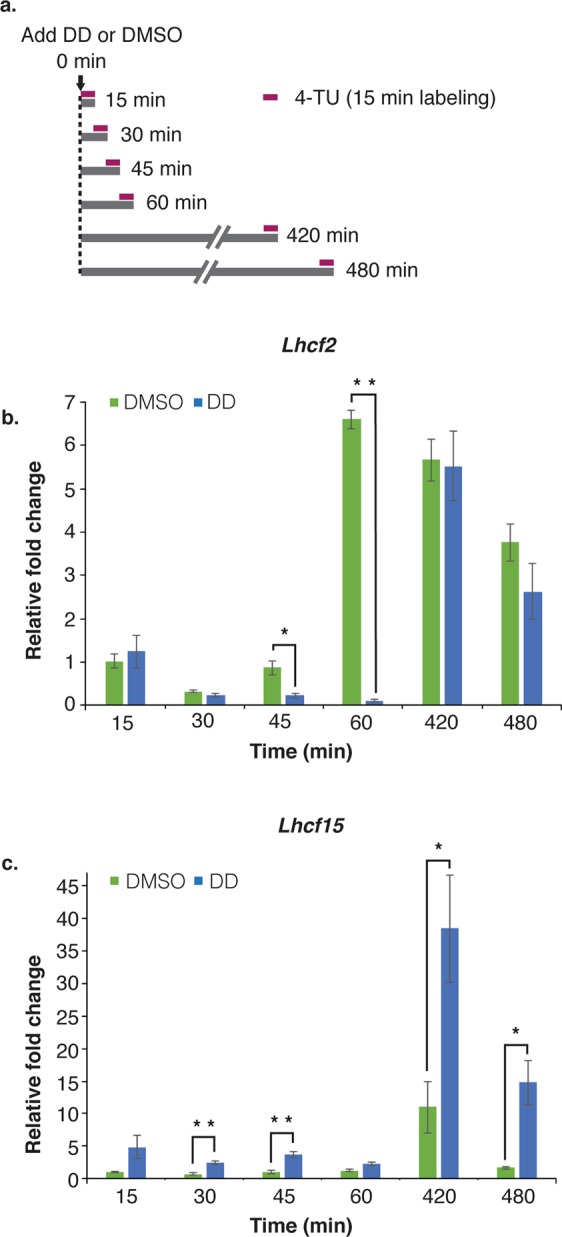
Varying *Lhcf2* and *Lhcf15* relative transcription rates after DD treatment. **(a)** Schematic representation of 4-TU short pulse (15 min, colored bar) experiment. Cultures received DD or DMSO at 0 min and were harvested at the indicated times. RT-qPCR analyses to determine relative transcription rates of newly synthesized *Lhcf2* mRNA **(b)** *Lhcf15* mRNA **(c)** after DD and DMSO treatments. Cell density 2.5 × 10^6^ cells/ml, n = 3 biological replicates. Reference gene was *TBP* (TATA-box binding protein). Relative transcription rates are by comparison with the 15 min time point for DMSO solvent control. Relative fold change = mean ± SEM. *P < 0.05, **P < 0.01, ***P < 0.001, two-tailed student’s t-test.

A short 4-TU pulse labeling experiment was carried out to determine *Lhcf2* and *Lhcf15* transcription rates in DD and DMSO treated cultures. The advantage of short pulse labeling is that the labeled newly synthesized RNA fraction will include actively synthesized mRNA pool from the nucleus; thus, reducing chances of mRNA degradation^45^. We designed short pulses of 0.5 mM 4-TU for 15 minutes for various time intervals after DD or DMSO treatments (Fig. 4a). Although both synthesis and degradation of labeled newly synthesized RNA will occur during the 15 min labeling, transcription effects were considered predominant as *Lhcf2* and *Lhcf15* mRNA half-lives were long relative to this time frame, ranging from 1.5 h and longer. The results will be discussed as effects on transcription. In the pulse experiment, the fold changes in the RT-qPCR analyses were relative to the first time point of the DMSO control (15 min).

In the DMSO treated cells, *Lhcf2* transcription rate was initially reduced to 0.3 fold at 30 min, 0.87 fold at 45 min and increased to 6.6 fold at 60 min (Fig. 4b). In the later period of the light cycle, transcription was 5.6 fold at 7 h and 3.7 fold at 8 h. In the DD treated cells, *Lhcf2* transcription showed similar initial reduction of 0.24 fold at 30 min. However, *Lhcf2* transcription suppression continued and intensified (0.22 fold at 45 min and 0.1 fold at 60 min). In the later periods of light cycle, this inhibition entirely recovered and rate of transcription increased to 5.5 fold at 420 min (7 h) and to 2.63 fold at 480 min (8 h).

The rate of transcription of *Lhcf15* in the DMSO treated cells remained similar up to 1 h then increased at 7 h (11 fold) and 8 h (1.7 fold) (Fig. 4c). The rate of transcription of *Lhcf15* gene in the DD treated cells rapidly upregulated to 4.8 fold within 15 minutes and remained significantly high (2.3–3.7 fold) up to 1 h. For the later period, the synthesis of *Lhcf15* transcript significantly increased to 38.4 fold at 7 h and 14.7 fold at 8 h. In summary, DD exerted major and differential effects on *Lhcf2* and *Lhcf15* transcription rates that varied over the course of the experiment. These effects support transcription regulation as a major factor affecting the observed DD induced steady-state mRNA kinetics.

## Discussion

In our study of *P. tricornutum* responding to sublethal decadienal (DD), transcriptome analysis revealed early differential *LHC* mRNA regulations. Two divergently regulated members of the predominantly suppressed *LHCF* group of mRNAs encoding light harvesting antennae were further examined at the transcription and mRNA stability levels using an *in vivo* metabolic labeling approach. The kinetics and direction of transcription and mRNA stability changes were well correlated with the observed steady state up- and downregulations of these transcripts in response to DD treatment. We hypothesize various benefits of the observed regulations under both normal and sublethal DD conditions and make comparisons with stress regulation of photosynthetic genes in higher plants and other algae.

Transcriptome analysis of *LHC* genes was undertaken at 3 and 6 h of DD and solvent control treatments, which is within the general time period of previously described early DD effects on cellular components and physiological processes. The *LHCF* family was overwhelmingly characterized by mRNA suppression at 3 h which partially attenuated by 6 h. Strong suppressions of *LHC* mRNAs have also been observed in numerous studies including *P. tricornutum* under high light^7^ and nitrogen stress^46^ and in *Brachypodium distachyon* under cold stress^8^. A set of 4 unclassified LHC mRNAs and most detected *LHCR* transcripts were also suppressed and showed a pattern of recovery at the 6 h time point. A smaller group of transcripts were upregulated and characterized by increasing magnitude at 6 h compared to 3 h of DD treatment. These were *Lhcf15*, *Lhcr9*, *Lhcr*10 and all 3 detected *LHCX* family transcripts (*Lhcx1*, *Lhcx3*, *Lhcx4*). LHCR proteins are primarily associated with Photosystem I in diatoms where there is evidence for their function in both light harvesting and photoprotection, although specific protein functions have not been established^7,40^. Using reduced *Lhcx1* expression transgenic lines, LHCX1 has been demonstrated to be a major high light stress activated LHC which provides photoprotection through nonphotochemical quenching (NPQ)^47^. In addition, other LHCX proteins were correlated with contributing to NPQ function during nutrient deficient growth conditions and high light conditions^48^. We hypothesize that a photochemistry imbalance arises during the early response to DD and that increased abundances of specific LHCR and LHCX proteins may function to mitigate this imbalance. Taken together, the transcriptome analysis provides important new data on the kinetics and number of *LHC* transcripts which expands on the previous EST database of sublethal DD effects on gene expression^32^.

The response patterns for *LHCF* transcripts suggested different regulatory pathways and functional roles during the early DD response period. Downregulated *Lhcf2* and upregulated *Lhc15* mRNAs were chosen for finer resolution of their regulations and mechanisms of transcript level control. In the normal growth condition with a diurnal cycle, *Lhcf2* mRNA was highly light regulated. After an initial decrease from the 0 h level in the first hour, it gradually increased through the 6 h monitoring period. Overall, these kinetics are similar with a previously reported pattern in normal light intensity diel cycle^36^ and are consistent with positive light responsive elements in the *Lhcf2* promoter^36,37^. Exposure of cells to sublethal DD condition strongly reduced *Lhcf2* mRNA levels for the first 2 h followed by mRNA accumulation through 6 h in a similar pattern as the solvent control but at reduced levels. Several abiotic conditions, high light and nutrient starved, have also decreased *Lhcf2* mRNA levels^7,46,49^. In control cells, *Lhcf15* mRNA was reduced or comparable to the 0 h level through 4 h then showed a very modest increase at 6 h (1.7 fold). DD treatment stimulated *Lhcf15* mRNA levels. The major effect occurred after 4 h resulting in about a 14 fold increase at 6 h. Increased gene expression of *Lhcf15* was also shown for high light, prolonged dark and red light with varying kinetics^7,14,38^.

The transcription and/or mRNA stability regulation processes influencing the observed mRNA regulations in the early response to DD were unknown. We modified and applied a metabolic labeling procedure^50^ that labeled newly synthesized RNA *in vivo* with 4-TU. Experiments to measure transcription and mRNA decay employed 4-TU pulses and 4-TU pulse-chases, respectively, and monitored transcripts in newly synthesized RNA pools. For mRNA decay measurements, this method avoided toxicity associated with transcription inhibitory drugs used in several past *LHC* mRNA decay studies^8,34^.

In the solvent control, *Lhcf2* transcription was lower through 45 min compared to the level in the initial 15 min pulse. The 60 min pulse revealed a reversal by a strong increase in transcription which remained high but was declining at the 7 and 8 h time points. In the DD treated cells, transcription instead remained significantly lower at both the 45 and 60 min time points and only showed increases similar to the control cells at 7 and 8 h time points with also a declining trend. In contrast, *Lhcf15* transcription remained very low in the control cells until a modest increase was observed at 7 h. In DD treated cells, *Lhcf15* transcription was significantly higher in several early time points but a much larger relative transcription was measured at 7 h and remained higher than the solvent control at 8 h, with both declining. The observed kinetics for endogenous *Lhcf2* gene transcription activity in control cells are in general agreement with previous work probing transcriptional control mechanisms using transgenes. *Lhcf2* promoter-diatom phytochrome coding region transgene in *P. tricornutum* provided evidence of light activation and dark repression of the promoter^36^. Similarly, an *Lhcf2* promoter-GUS transgene showed a pattern of increasing then decreasing activity during 3–8 h after light onset^37^ and a *Lhcf2* promoter-luciferase transgene exhibited light-dark alteration in expression^51^.

Transcript levels due to altered transcriptional activities of the two genes were augmented in a complementary fashion by changes in their mRNA stabilities. Control cells showed variable *Lhcf2* mRNA half-lives during the light cycle. While a shorter 3 h pulse-chase showed a 7.5 h half-life, a longer 9 h pulse-chase showed a 1.5 h half-life. The DD treated cells exhibited a single half-life around 2.2 h regardless of the pulse-chase intervals. *Lhcf2* mRNA half-life decreased in the first 3 h after DD treatment (2.2 h) and remained shorter in the later phase of the light cycle when the control cells also had reduced the mRNA half-life. Thus, both control and DD treated cells made use of varying transcription and mRNA stabilities to impact *Lhcf2* mRNA levels. DD treatment reduced transcription within the first hour and reduced mRNA stability within the first 3 h followed by later closer convergence to transcription in control cells. In contrast, the main period of *Lhcf15* mRNA increase was after 3 h of DD treatment. This late regulation was contributed by a higher rate of *Lhcf15* transcription after the first hour compared to control and a longer mRNA half-life after 3 h of DD treatment compared to the control cells which exhibited a shorter half-life in the late period. In summary, while it is also common for an organism to largely use transcription or mRNA stability regulation to achieve a change in mRNA levels, *P. tricornutum* has used both mechanisms to efficiently achieve varying *Lhcf2* and *Lhcf15* mRNA levels during normal and sublethal DD conditions. Destabilization and stabilization of *Lhcf2* and *Lhcf15* mRNAs, respectively, enables a more rapid adjustment of cytoplasmic mRNA levels and correspondingly potentially more rapid effects on translation than using only transcriptional control mechanisms. The observed widespread *LHCF* mRNA downregulations in DD treated cells at 3 h and partial restoration at 6 h together with the close chromosomal clustering of *Lhcf2* with several other *LHCF* genes and *LHCF* promoters having a number of shared motifs^51^ may indicate some common mechanisms. However, whether one or both mechanisms are employed for regulation of other *LHC* mRNAs described in this study will require further investigations. Several mechanisms have been documented in *P. tricornutum* which have been shown to affect transgene expression or likely function in transcription modulation including enhancers, transcription factors, gene methylation, and small RNA mediated DNA methylation^51,52^. Diatom specific novel miRNAs which may function in mRNA stability regulation were identified under various nutrient stresses where one of the potential targets was *Lhcr11* mRNA^53^.

What is the functional significance of the *LHC* mRNA regulations by sublethal DD treatment in the early response period? A major consideration is whether the mRNA changes lead to significant protein abundance changes and physiological effects on photosynthesis. For the transiently downregulated *LHC* mRNAs, reduced protein synthesis may not substantially impede photosynthetic processes as existing data from higher plants, albeit limited, has shown LHC proteins generally had low turnover rates in normal conditions^54^. Based on widespread abiotic and biotic stress induced photosynthesis-related mRNA suppression and the hypothesis that photosynthesis processes can be maintained well for a period despite reduced synthesis of component proteins, several studies have advanced hypotheses that the principal benefit is conservation of energy and material resources such as nucleotides, amino acids, ATP, and ribosome capacity so that they are deployed for stress-related processes to achieve acclimation^2,6^. Diatoms have shown versatile and rapid shifts in metabolic processes in changing environments^55^. Previous studies showed that new metabolic demands and changes in cell survival fitness manifested within the first few hours of sublethal DD treatment^26,31^ in *P. tricornutum* while the cells also maintained stable photosynthetic efficiency^33^. In sublethal DD treated *P. tricornutum* cells, the observed reduced growth rate for the first 3 days would also likely enable more resources for acclimation^31^. The present study showed that DD-induced *LHC* mRNA suppressions (and likely other mRNAs) were early and transient, and primarily occurred within the first 3 h. We believe that these findings taken together support the hypothesis that the observed *LHC* gene suppression enables conservation of energy and materials as part of acclimation.

For *LHC* mRNAs upregulated by sublethal DD treatment, their mRNAs increased over the 6 h DD treatment period studied. Clear precedents exist for stress condition stimulation of LHC protein abundance for photoprotection such as LHCX family members in *P. tricornutum*^48^. LHCF15 protein was not detected under normal growth conditions but accumulated after several days under red light enriched conditions to form an additional antennae assembly^39^. LHCF15 proteins display a strongly red-shifted absorption when in oligomeric complexes and were shown to function in light harvesting to PSII with also evidence of PSI association, the latter similar to the well-known red-shifted proteins that form part of PSI antennae in higher plants^40,56^. Red-shifted antennae proteins have been observed especially in cyanobacteria and microalgae and they have been postulated to enable niche expansion by accessing a less utilized portion of the light spectrum for photosynthesis^57^. In ecological value, LHCF15 was postulated to confer expanded light absorption to permit growth in shaded locations depleted of main photosynthetic wavelengths by other photosynthesizing organisms in the water column or in a biofilm on a solid coastal substrate^39^. In sublethal DD conditions, we speculate that the small set of *LHC* mRNAs which accumulate during the early response result in higher levels of encoded proteins in support of photosynthesis and cell health. Specifically, LHCF15 accumulation may function in expanding the useable light spectrum, LHCR9 and LHCR10 may increase PSI antennae cross section and the induced LHCXs may increase photoprotection. Future work to investigate the potential development of these acclimation processes may help understand the concerted use of multiple mechanisms to benefit cell survival in regions of herbivory or nutrient depletion stress with varying oxylipin levels.

## Methods

### Culture conditions and treatments

Axenic culture of *Phaeodactylum tricornutum* Bohlin (CCMP2561) was obtained from National Center for Marine Algae and Microbiota, Bigelow Laboratory for Ocean Sciences, USA. Cultures were grown in autoclaved f/2 media made with filtered seawater from the Gulf of Mexico under cool white fluorescent light (90–100 µmol m^−2^s^−1^) at 20 °C in a 12 h light: 12 h dark cycle. For all experiments, batch cultures were mixed for homogeneity and divided into individual flasks as biological replicates on the day before treatments. *Trans*,*trans* −2,4-decadienal (DD, W313505) and 4-thiouracil (4-TU, 440736) stocks were freshly prepared in anhydrous DMSO solvent (276855) (Millipore Sigma). Each biological replicate was treated with 10 µM DD or 0.075–0.1% DMSO solvent control at 2.5–3 h after the onset of the light cycle. All treatments and cell harvests were performed during the light period.

### RNA extraction, library preparation and sequencing analysis

Cultures were harvested by vacuum filtration (Whatman Nuclepore Track-Etched 0.4 µM filters). Cells on filters were frozen by liquid nitrogen and stored in −80 °C. Samples were resuspended in PureLink Plant RNA Reagent (12322012 ThermoFisher) and mechanically disrupted in QIAGEN Tissue Lyser^LT^ (2 × 2 min, 1 × 1 min, 30 sec pauses, 50 oscillations/sec) with Zirconia-silica beads (0.5 mm, Biospec) according to Tissue Lyser^LT^ instructions. Total RNA was purified according to the PureLink Plant RNA Reagent protocol. Samples were assessed by Nanodrop (ThermoFisher) spectroscopy, denaturing RNA gels and Bioanalyzer (Agilent). RNA yields ranged from 10–20 µg and 60–100 µg total RNA per 50 and 250 ml cultures, respectively. For RNA-seq analysis, cells (3–3.2 × 10^6^ cells/ml) were treated with 10 µM DD or 0.1% DMSO for 3 and 6 h, two biological replicates per treatment. Total RNA samples were submitted to the Genomic Sequencing and Analysis Facility at the University of Texas at Austin. RNA quantity and quality were assessed using a Bioanalyzer (Agilent). Poly (A) RNA was enriched using Poly (A) Purist Magnetic Kit according to manufacturer’s instructions (ThermoFisher) with samples processed twice for higher enrichment. Library preparation used the NEBNext Module dUTP directional RNA method according to the manufacturer’s instructions (New England Biolabs). RNA sequencing was performed using Illumina NextSeq 500 platform, SR75 run type. For mapping reads to the *Phaeodactylum tricornutum* reference, GFF filtered models and genome files available at Ensembl (http://protists.ensembl.org/Phaeodactylum_tricornutum/Info/Index/) were used. Mapping and assembly were analyzed with TopHat (https://ccb.jhu.edu/software/tophat/index.shtml). Differentially expressed genes were determined by negative binomial distribution method using DESeq2 bioconductor package with a DESeq false-discovery adjusted p-values < 0.05. Gene expression heat map generated through the web tool Heatmapper (http://www2.heatmapper.ca/expression)^58^.

### 4-TU labeling, biotinylation and fractionation of newly synthesized RNA

Total RNA samples, each from 200 ml cultures, were DNase (EN0521 ThermoFisher) treated and biotinylated as described^50^ with the following modifications. After DNase treatment, to reduce genomic DNA and protein contamination, samples were extracted with equal volumes of Acid-Phenol: Chloroform (AM9720 ThermoFisher), organic phases were back extracted with 10 mM Tris, 0.1 M NaCl, the combined aqueous precipitated with 2-propanol, pellets were washed with 75% ethanol and resuspended in RNase free water. RNA samples (equal masses, usually 40–60 µg) were biotinylated (Biotin-HPDP, ThermoFisher) in 2 ml screw cap microfuge tubes placed vertically in a shaker (250 rpm) for 1.5 h at room temperature in dark. After the reactions, the following steps were implemented to reduce biotin-HPDP contaminations. Samples were extracted with chloroform:isoamyl alcohol (24:1) with added back extraction steps and the aqueous phases were separated using MaXtract High Density phase lock tubes (129056 QIAGEN). After alcohol precipitation and resuspension as above, samples were passed through Sephadex G-50 columns (11274015001, Millipore Sigma) and alcohol precipitated and resuspended. Biotinylated RNA samples (equal masses) were subjected to µMACS columns to capture newly synthesized RNA fractions as described by µMACS Streptavidin Kit protocol (130–074–101 Miltenyi Biotec) with minor modifications. Pre-existing RNA fractions were separated with 6 × 900 µl wash buffer (5 × 65 °C, 1× room temperature). After the last wash, newly synthesized RNA fractions were recovered with 2 × 200 µl of freshly prepared 100 mM DTT (D-9779 Millipore Sigma) applied in 5 min intervals. Combined fractions were then mixed with 2 µg glycogen, RNA grade (R0551, ThermoFisher) and precipitated with equal volumes of 2-Propanol. Pellets were washed with 75% Ethanol and resuspended in 38 µl DEPC treated water. Mass of newly synthesized fractions were assessed by Agilent Bioanalyzer with an Agilent RNA 6000 Pico kit.

### Reverse transcription and RT-qPCR

Total RNA samples (1 µg) were DNase treated according to DNase I, RNase-free protocol (EN0521 ThermoFisher) and reverse transcribed according to High-Capacity cDNA Reverse Transcription Kit protocol (4368814 ThermoFisher). For the newly synthesized RNA fractions, 16 µl RNA aliquots were used in doubled size DNAse and cDNA synthesis reactions as above. For RT-qPCR, primer sets were designed for each target gene using PerlPrimer software (perlprimer.sourceforge.net). Standard curves were prepared over 5 or 6 log_10_ concentrations of cDNA template. Acceptable primers (Supplementary Table S1) showed 90–110% amplification efficiency with slopes of −3.2 to − 3.6 with R^2^ values of 0.99 or higher.

For total RNAs, RT-qPCR reactions were performed according to SYBR Green PCR Master Mix protocol (4309155 ThermoFisher) using ThermoFisher ViiA7 Real-Time PCR System instrument and standard conditions (2-cycle PCR, 40 cycles of 95.0 °C for 15 sec and 60.0 °C for 1 min). For newly synthesized RNA fractions, each reaction was performed with 2 µl directly from the 40 µl cDNA synthesis reaction and 18 µl of primers and KAPA SYBR FAST qPCR Master Mix (KK4620 Kapa Biosystems) and run fast qPCR cycle according to the manufacturer’s protocol (KK4620 Kapa Biosystems; 40 cycles of 95 °C 3 seconds and 60 °C for 20 seconds). The default settings for threshold cycle (C_t_) determination were used. All samples were run in triplicates. Relative fold changes were calculated according to the 2^-∆∆CT^ method^59^. TBP (TATA-BOX binding protein) was validated as the stably expressed reference gene.

### 4-TU pulse experiment

For each set of biological replicates, 10 µM DD and 0.075% DMSO were added in a staggered manner following 2.5 h of light cycle. Each biological replicate was treated with 0.5 mM 4-TU as final concentration at 15 min before harvest. RNAs were extracted, biotinylated, purified and newly synthesized fractions used in cDNA synthesis and RT-qPCR reactions as described above. Relative fold changes were calculated as above.

### 4-TU pulse-chase experiments

For all 4-TU pulse-chase experiments, each set of biological replicates were treated with 0.5 mM 4-TU at 45 min after initiation of the light cycle for 1.5 h. Cultures were harvested through vacuum filtration. Cells were resuspended in the same volumes of fresh f/2 media as chase with immediate application of 10 µM DD or 0.075% DMSO in a staggered manner. RNAs were extracted, biotinylated, purified and newly synthesized fractions used in cDNA synthesis and RT-qPCR reactions as described above. Relative fold changes were calculated according to the 2^−CT^ method^60^. Half-lives of mRNAs were determined from the decay constants^61^.

## Data Availability

The RNA-seq datasets and accompanying information are available at Gene Expression Omnibus (GEO) genomics data repository as a GEO DataSet (GEO accession GSE142157) (https://www.ncbi.nlm.nih.gov/geo/query/acc.cgi?acc = GSE142157).

## Supporting Information

Supporting Information.
Supporting Information 1.
