# Supplementary material for: Early dynamics of photosynthetic Lhcf2 and Lhcf15 transcription and mRNA stabilities in response to herbivory-related decadienal in Phaeodactylum tricornutum
Source: Sci Rep. 2020 Feb 6;10:2029. doi: 10.1038/s41598-020-58885-9 (PMC7005025; doi:10.1038/s41598-020-58885-9)
Supplement: Supplementary file 1 — Supporting Information. [file 41598_2020_58885_MOESM1_ESM.pdf]

Supplementary Information for

Early dynamics of photosynthetic *Lhcf2* and *Lhcf15* transcription and mRNA stabilities in response to herbivory-related decadienal in *Phaeodactylum tricornutum*

S. Islam, T. Sabharwal, S. Wu, TJ Bullock and M. C. Mehdy

Department of Molecular Biosciences, University of Texas at Austin, USA

Figure S1: *P. tricornutum* culture growth with 4-TU……………………………………………………………………..…2

Figure S2: Dot blot analysis to assess 4-TU incorporation in the biotinylated total RNA fractions…3

Figure S3. Effects of uracil chase on cell color and rate of cell harvesting by filtration. …………4

Figure S4: RT-qPCR analyses of *Lhcf2* and *Lhcf15* mRNA levels from 4-TU labeled biotinylated total RNA fractions in the pulse-chase experiments (Figure 3) ……………………..……………………………………………5

Figure S5. Semi-log plots of newly synthesized RNA fractions concentrations versus time for pulse-chase experiments (Figure 3)………………………….………………….……………………………………………………………6

Table S1: Genes and primers used in RT-qPCR……………………..………………………………………………..….7

Table S2. Live cell percentages measured by Evans blue cell viability tests with 0.5 mM 4-TU or 0.1% DMSO treatments for 1.5 and 3 h. ……………………………………………………………………………………………8

a

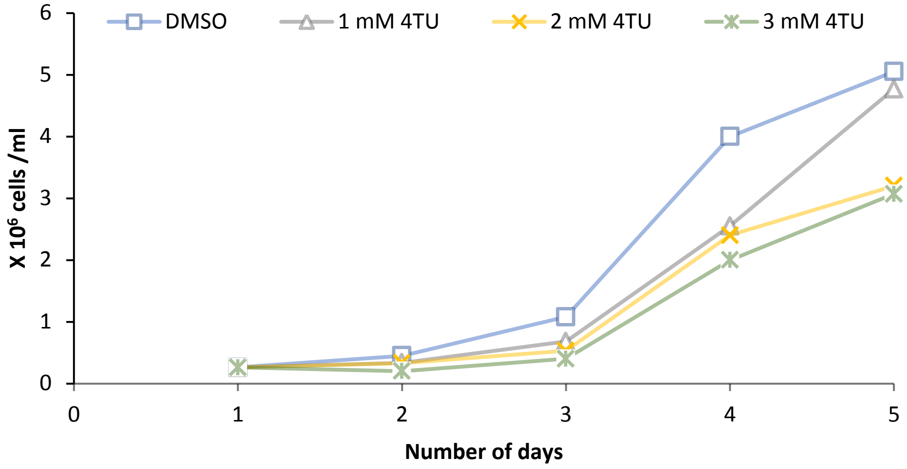

b

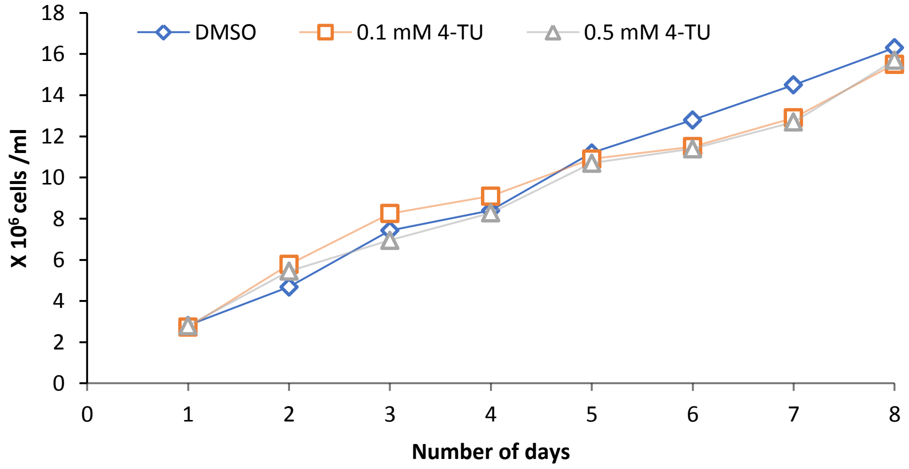

Figure S1: ***P. tricornutum*** **culture growth with 4-TU. a.** Cell cultures received 1 mM, 2 mM, 3 mM 4-TU or 0.1% DMSO as final concentrations at day 1. **b.** Cell cultures received 0.1 mM, 0.5 mM 4-TU or 0.1% DMSO as final concentrations at day 1. Cell densities were determined with a hemocytometer (Reichert Co.) n = 3 biological replicates.

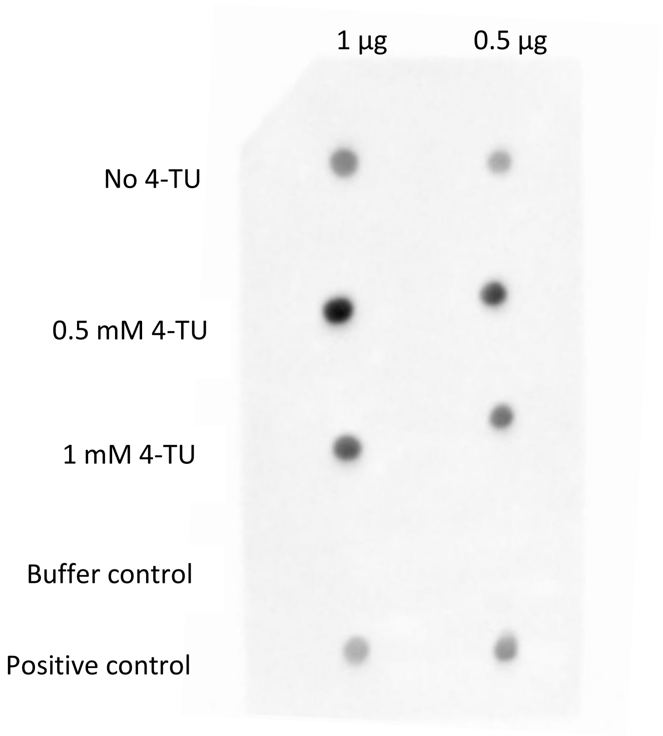

Figure S2: Dot blot analysis to assess 4-TU incorporation in the biotinylated RNA fractions. Cultures received 0.5 mM, 1.0 mM 4-TU or 0.1% DMSO solvent control as no 4-TU for 1.5 h. Cell densities were 2-2.5 X10^6 cells/ml. Purified RNA samples were subjected to biotinylation reactions. Biotinylated RNA fractions from the experiment and a positive control from 0.5 mM 4-TU treated cells in a previous experiment were spotted on Zeta-Probe Membrane (1620165 Bio-Rad) in two dilutions as well as the buffer control. Dot blot was performed as described [49] with minor modifications. Membrane was probed with 1:5000 dilution of 1 mg/mL Streptavidin-horseradish peroxidase (M00089 GenScript). The biotin-streptavidin signal was detected according to the manufacturer's protocol of Pierce ECL Western Blotting Substrate (32209 ThermoFisher) using G:BOX Chemi XX9 - high resolution gel imaging system (Syngene) using default settings. Figure shows full size image of one representative blot, exposed to optimize for gray scale.

a **b**

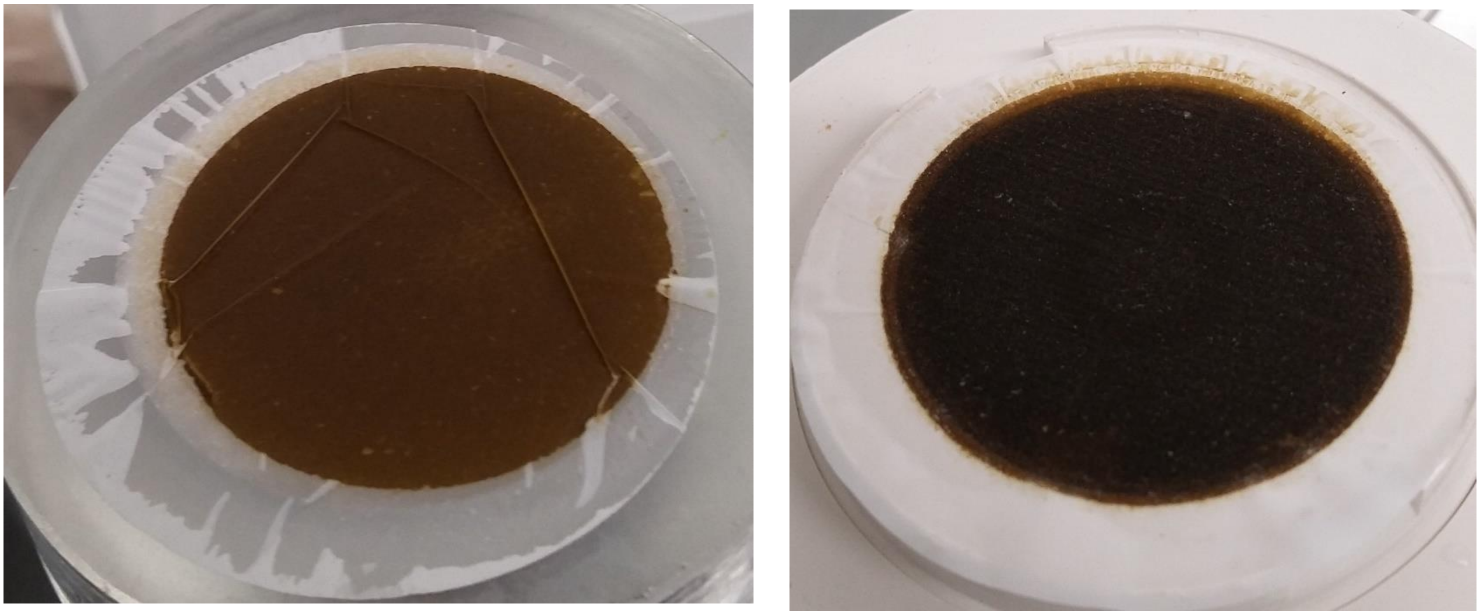

Cells without Uracil

(2-3 min harvest time)

Cells with 10 mM Uracil

(6-9 min harvest time)

Supplementary Figure S3. Effects of uracil chase on cell color and rate of cell harvesting by filtration. Cultures (200 ml) were treated with 0.5 mM 4TU for 1.5 h then transferred to f/2 media or f/2 media containing 10 mM uracil. Figure shows representative images of cells collected on filters after 1 h incubation in f/2 media (**a**) or f/2 media containing 10 mM uracil (**b**). Times required to harvest cells are given below each label.

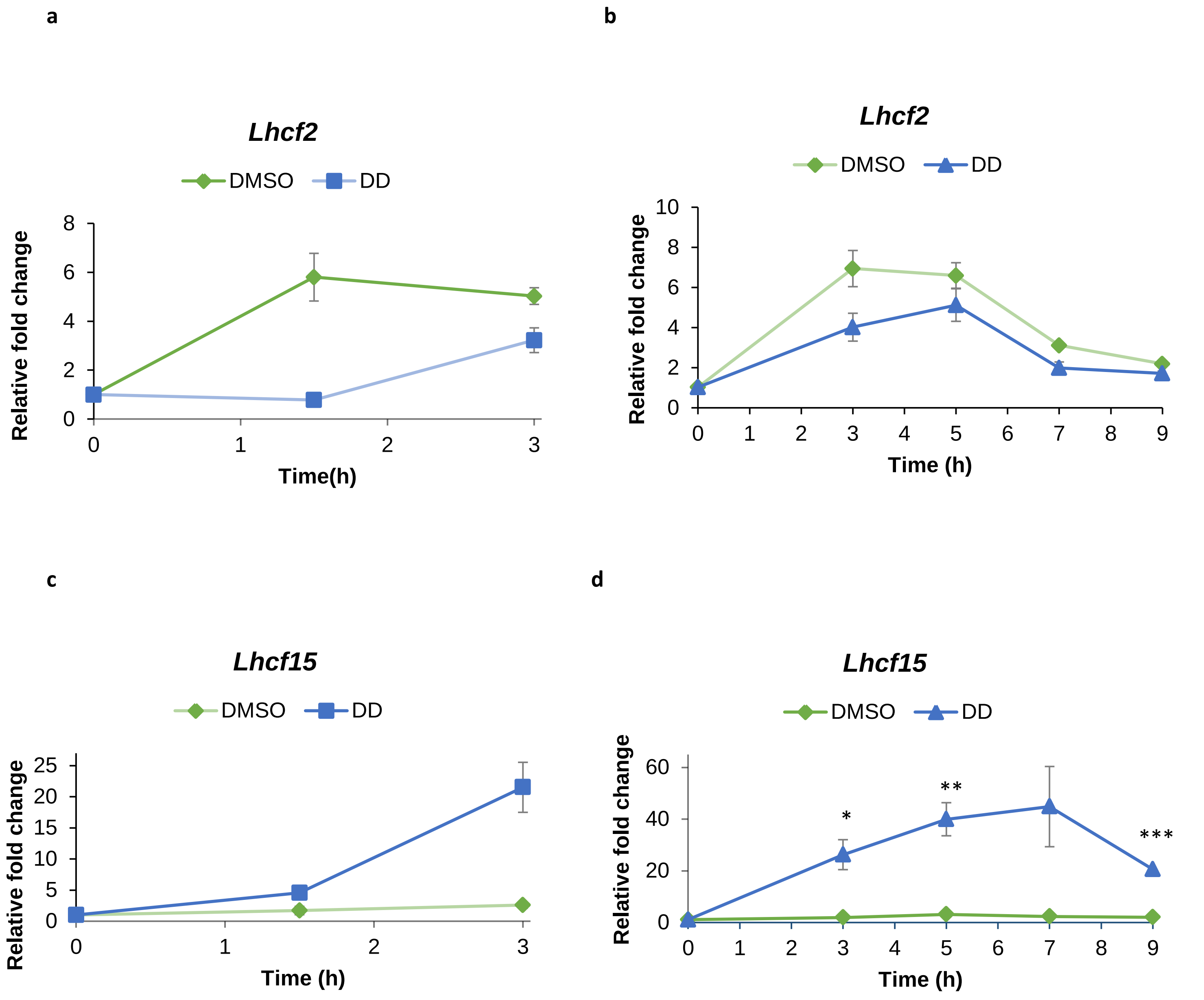

Figure S4: RT-qPCR analyses of *Lhcf2* and *Lhcf15* mRNA levels from 4-TU labeled biotinylated total RNA fractions in the pulse-chase experiments (Figure 3). DD or DMSO were added at 0 h. RT-qPCR analyses of *Lhcf2* mRNA kinetics for 3 **(a)** and 9 h **(b)**. RT-qPCR analyses of *Lhcf15* mRNA kinetics for 3 **(c)** and 9 h **(d)**. Cell density 2.5X 10^6 cells/ ml, n=5 biological replicates. Reference gene was *TBP* (TATA-box binding protein). Fold changes are relative to 0 h. Relative fold change = mean ± SEM. Error bars not appearing are merged into the time points. *P< 0.05, **P< 0.01, ***P< 0.001, two-tailed student's t-test

a **b**

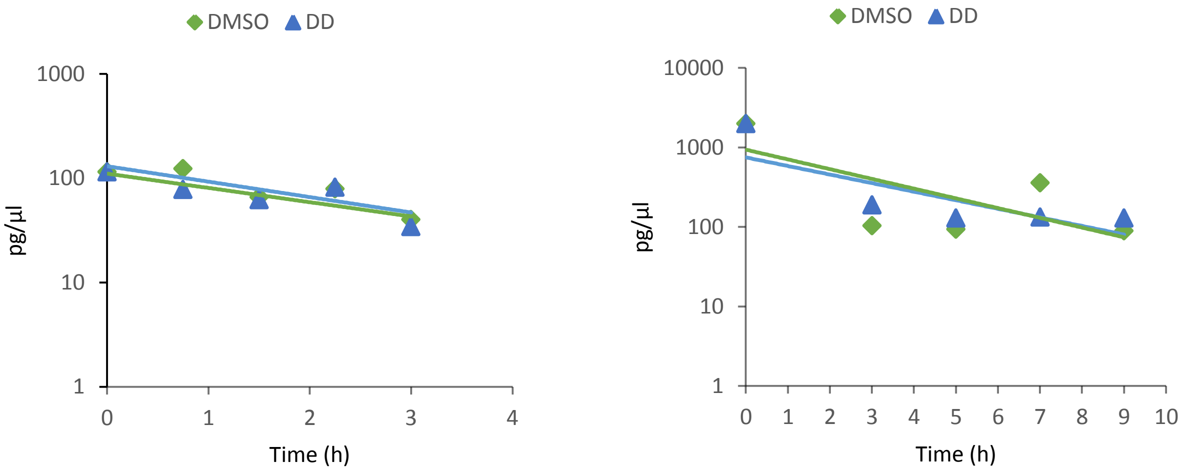

Figure S5. Semi-log plots of newly synthesized RNA fractions concentrations versus time for pulse-chase experiments (Figure 3). Bioanalyzer quantified newly synthesized mRNA fractions concentrations (equal volumes of elution) that resulted from equal masses of biotinylated RNA fractions. Concentrations of newly synthesized mRNA fractions (on log scale as Y axis) for 3 h **(a)** and 9 h **(b)** pulse-chase experiments from DD and DMSO treated cultures. n = 3 biological replicates.

Table S1: Genes and primers used in RT-qPCR.

Gene name	NCBI ref.	Forward Primer	Reverse Primer
Lhcf2	XM_002177834.1	5'-ATATTGACTTTGGATGGGACTCCT-3'	5'-TTGACTCCCAACTGTTCGTG-3'
Lhcf9	XM_002183673.1	5'-GCGTAACAACTACATTGACTTTGG-3'	5'-GTTGCTCGTGGACCATAAGG-3'
Lhcf10	XM_002182183.1	5'-CCAAGTTCACAGACATTCCTG-3'	5'-CAAAGGTATCCCAACCAAAGTC-3'
Lhcf11	XM_002184583.1	5'-CTGTCGTTGGTTACCTTGTCC-3'	5'-CCAGTTATGTCCTTCATGACCG-3'
Lhcf15	XM_002183345.1	5'-GGATGAGGAAACCATGAACAC-3'	5'-GTCAAAGCCATCAAAGCCAG-3'
TBP	XM_002186285.1	5'-TCCAGTACTGTAAATCTAGGCAC-3'	5'-TAATCGCATGATTACGGCAC-3'

Table S2. Live cell percentages measured by Evans blue cell viability tests with 0.5 mM 4-TU or 0.1% DMSO treatments for 1.5 and 3 h. Cell viabilities were assessed using Evans Blue (E2129-10G, Millipore Sigma) [62]. Cultures were treated with 0.1% DMSO or 0.5 mM 4-TU. Biological replicates (5 ml) were withdrawn at 1.5 and 3 h and Evans Blue dye (1% stock solution) was added as 0.02% final concentration. Cultures were incubated for 15 minutes prior to live-dead cell count by a hemocytometer under light microscope. Unstained cells were considered as live cells and deep blue stained cells were considered as dead cells because of Evans Blue dye uptake. An average of triplicate cell counts was taken as final count for each biological replicates. Data were converted to % unstained cells. Values given are the mean of 3 biological replicates. Cell densities were 2-2.5 X10^6 cells/ml.

Time (h)	0.1% DMSO	0.5 mM 4-TU
1.5	99.94	99.9
3	99.04	99
